# Multifaceted empathy differences in children and adults with autism

**DOI:** 10.1038/s41598-021-98516-5

**Published:** 2021-09-30

**Authors:** Jennifer M. Quinde-Zlibut, Zachary J. Williams, Madison Gerdes, Lisa E. Mash, Brynna H. Heflin, Carissa Cascio

**Affiliations:** 1 Vanderbilt Brain Institute, Vanderbilt University, Nashville, TN USA; 2 Frist Center for Autism and Innovation, Vanderbilt University, Nashville, TN USA; 3 Medical Scientist Training Program, Vanderbilt University School of Medicine, Nashville, TN USA; 4 Department of Hearing and Speech Sciences, Vanderbilt University Medical Center, Nashville, TN USA; 5 Graduate Program in Criminology and Justice Policy, Northeastern University, Boston, MA USA; 6 San Diego Joint Doctoral Program in Clinical Psychology, San Diego State University/University of California, San Diego, CA USA; 7 Graduate Program in Clinical Psychology, Florida International University, Miami, FL USA; 8 Department of Psychiatry and Behavioral Sciences, Vanderbilt University Medical Center, Nashville, TN USA; 9 Vanderbilt Kennedy Center, Vanderbilt University, Nashville, TN USA

**Keywords:** Social behaviour, Social neuroscience, Empathy

## Abstract

Although empathy impairments have been reported in autistic individuals, there is no clear consensus on how emotional valence influences this multidimensional process. In this study, we use the Multifaceted Empathy Test for juveniles (MET-J) to interrogate emotional and cognitive empathy in 184 participants (ages 8–59 years, 83 autistic) under the robust Bayesian inference framework. Group comparisons demonstrate previously unreported interaction effects between: (1) valence and autism diagnosis in predictions of emotional resonance, and (2) valence and age group in predictions of arousal to images portraying positive and negative facial expressions. These results extend previous studies using the MET by examining differential effects of emotional valence in a large sample of autistic children and adults with average or above-average intelligence. We report impaired cognitive empathy in autism, and subtle differences in emotional empathy characterized by less distinction between emotional resonance to positive vs. negative facial expressions in autism compared to neurotypicals. Reduced emotional differentiation between positive and negative affect in others could be a mechanism for diminished social reciprocity that poses a universal challenge for people with autism. These component- and valence- specific findings are of clinical relevance for the development and implementation of target-specific social interventions in autism.

## Introduction

Autistic individuals have long been erroneously presumed to lack the ability and/or desire to relate to others^1^. Currently, a clinical diagnosis of autism is characterized by marked social and communication deficits, along with restrictive and repetitive behaviors^2^. Extant theories on the origins of these autistic traits focus on genes^3^, brain activity^4^, and psychological features^5^. The ‘Theory of Mind’(TOM) account of autism^5^, proposed that autism is characterized by a core deficit in the understanding of others’ intentions, knowledge, and emotions. The field of autism research has largely moved beyond such “core deficit” models^6–8^ in favor of more holistic models that address the complexities of interactions between individuals with and without autism. However, the TOM theory set the tone for much of the early empathy research in the autistic population. Considered a social ‘glue’ that allows for successful human relationships, empathy is an essential component of social interactions with which individuals on the autism spectrum reportedly struggle^9–11^.

Various studies investigating empathy differences in autism have reported conflicting results, including intact empathic physiological responses^12,13^, but reduced or dampened self-reported empathy^13,14^. Notably, empathy research has been slow to develop a well-defined operationalized definition of “empathy” or its various components, and there remains no global consensus on best practices for the measurement of this construct^15,16^. Thus, early autism studies employing differing approaches to measure this inherently complex neuropsychological concept often reported findings that were not always based on the same empathy subconstructs^11,17^.

Empathy has traditionally been considered to include the distinct constructs of **emotional empathy**, the ability to *share* another person’s feelings and **cognitive empathy**, the capacity to *understand* such feelings^18,19^. A third, now increasingly differentiated component, **empathic concern,** has recently gained consideration as part of the multifaceted nature of empathy^20–22^. Empathic concern is characterized by greater *others*-oriented relational mentalities and encompasses emotional sentiments (e.g. sympathy and compassion) towards someone else’s experience^11^. Though the distinction between emotional empathy and empathic concern is subtle, emotional empathy describes *sharing* another’s feelings, which involves *self*-orientation (i.e., “mirroring” emotion), while empathic concern does not require having the same feeling but being aware of and concerned about another’s feeling In fact, this capacity for self-other distinction is considered to be crucial and integral to the empathic experience^23^.

Many studies have now explored these 3 constructs both separately, and to a lesser extent, in some combination within a single autism sample. This is notable given the growing evidence that these processes typically work together to form a unified percept^23^. Thus it would be difficult, for example, to speculate on the underpinnings of emotional empathy differences and how these may relate to autism symptomatology, without assessing these separate but related constructs simultaneously. To this end, empathy differences between autistic and neurotypical (NT) individuals have been primarily assessed using multidimensional self-report tools like the Interpersonal Reactivity Index^24^ (IRI), Empathy Quotient^14^ (EQ), and Questionnaire of Cognitive and Affective Empathy^25^ (QCAE) (for a review, see Song et al., 2019). While these well-validated self-report measures have informative potential, they are not without limitations. Primarily, they are subject to social desirability biases^26^, and subjective variability in interpretation that can ultimately result in under- or over-reporting.

To address the limitiations of widely-used self-report measures, Dziobek and colleagues (2008) developed the Multifaceted Empathy Test (MET) as a performance-based measure of empathy. The MET is a computerized task that assesses both emotional and cognitive empathy in response to a series of emotionally charged facial expressions. According to the creators of the MET, this task is more ecologically valid than self-report measures of empathy, as it does not rely as heavily on the level of insight an individual has into their own emotions. The MET was also designed to mitigate potential social desirability biases in empathy ratings by asking participants to rate their level of arousal in response to each stimulus, which ostensibly serves as an implicit measure of emotional empathy. This measure has contributed meaningfully to the study of empathy in autism, as large effects of diagnostic group on the cognitive empathy subcomponent of the MET^26–28^ indicate that group differences in empathy demonstrated using questionnaire measures are not simply due to differences in emotional self-awareness or social desirability biases.

The ability to perceive and distinguish positive from negative emotional situations is an important feature of empathy and drives our tendency to engage in prosocial behaviors. This process is also mediated by the limbic system and allows for intentional and motivated reactions to our ever-changing environment^29^. Indeed, face perception studies suggest that both limbic neural activity^4,30,31^, and gaze patterns^32^ are differentially responsive to faces of negative versus positive emotion. For example, faces displaying negative emotion typically elicit stronger activation in the left amygdala^4^, a larger early negative component (N300)^30^, and increased scanning of the eyes^32^. Though there is precedent for a link between the amygdala and valence-specific emotion processing patterns in autism, the role of emotion in autism is not yet clear^33^. Further, the capacity to distinguish positive from negative emotions in others is important for the ability to produce appropriate resonant behaviors that ultimately help build social rapport^34^.

Although some prior work has indicated that emotional valence may moderate diagnostic group differences in empathy^27,35^, studies on this topic are scarce and inconsistent, and it remains unclear whether these differences relate to autism symptomatology. Studies directly comparing cognitive and affective empathy in response to positive and negative faces in autism reported impaired emotion recogniton of positive but not negative emotions^33,35^, while another reported emotional empathisizing differences in autism for negative but not positive emotions^27^. Notably, participant samples from these studies were predominantly male, providing little to add to reports of sex differences in empathy from theoretical^36^ and performance-based studies alike, and thus warrant further exploration and confirmation in single cohorts that include females.

## The present study

In light of this emerging but incomplete picture of empathy in autism, the present study explores empathy as a multifaceted construct that is potentially modulated by valence in individuals with autism. To extend previous findings, an age-appropriate adapted version of the MET, the MET-Juvenile^28^, consisting of 16 positive and 16 negative emotional images (decreasing testing time to accommodate younger participants), was administered to a large sample with a wide age range. Notably, though component-specific age effects on empathy have been reported^37,38^, there is no clear agreement on how these capacities may be related to maturation^11^. For example, while some studies suggest that empathy increases with age^37^, others suggest that older adults have lower empathic abilities^39^, see Sun et al., 2017 for a review^40^. Thus, participants from a broad age range were included in the present study thereby ensuring grounds for exploration of any age or age group related effects in empathy responses. Group differences in cognitive and emotional empathy were assessed globally in the context of the hypothesis that empathy is a complex, developmental process involving emotional and cognitive components that are moderated by stimulus valence.

## Results

### Demographic variables

In total, 184 participants were included in the analysis sample: 45 children and adolescents (35 male; mean age = 11.53) with autism, and 43 neurotypical children and adolescents (34 male; mean = 11.86), 38 adults with autism (21 male; mean age = 27.65), and 58 neurotypical adults (36 male; mean age = 32.69). The two diagnostic groups were approximately equivalent in terms of sex ratio (OR = 1.091, 95% CrI [0.583, 2.003], *BF*_10_ = 0.177), although they significantly differed with respect to age (*d* = 0.457 [0.146, 0.773], *BF*_10_ = 22.2), full-scale IQ (*d* = 0.532 [0.224, 0.846], *BF*_10_ = 94.4), VIQ (*d* = 0.657 [0.326, 1.010]), and SRS-2T-scores *(d* = − 2.896 [− 3.478, − 2.349]). Additionally, the NT group had numerically higher PIQ scores on average than the autistic group, although the Bayes factor indicated only “anecdotal” evidence in favor of a group difference (*d* = 0.284 [− 0.021, 0.588], *BF*_10_ = 1.59).

### Hierarchical Bayesian models

Model selection procedures indicated that cognitive empathy scores were best predicted by a model including all baseline predictors and VIQ score ($P\left({\mathcal{M}}_{BL+VIQ}|Data\right)=0.405$; *R*^2^_Bayes_ = 0.257 [0.242, 0.272]). The posterior inclusion probability of VIQ was relatively high (*P*_VIQ_ = 0.734), although the inclusion Bayes factor for VIQ did not meet the a priori threshold of 3 (*BF*_inc_ = 2.76). Inclusion Bayes factors also provided substantial evidence *against* the inclusion of all interaction terms, PIQ, and SRS-2 T-score as predictors of cognitive empathy (Table 1).

**Table 1:** Bayesian model analysis results.

Best fit predictor	OR [95% CrI]	BF_10_	BF_inc_	BF_ROPE_	P_ROPE_
**Cognitive empathy**					
Diagnosis (AUTISM)	**0.726 [0.587, 0.906]**	**6.854**		**3.677**	**0.00**
Sex (F)	1.113 [0.917, 1.352]	0.179		0.110	0.00
Age group (Child/Adolescent)	**0.604 [0.462, 0.795]**	**58.271**		**37.80**	**0.00**
Valence (Negative)	0.545 [0.267, 1.071]	1.633		1.609	0.00
Verbal IQ Z-score	1.126 [1.037, 1.218]	2.949	2.760	0.213	0.00

Best fit predictor	β [95% CrI]	BF_10_	BF_inc_	BF_ROPE_	P_ROPE_
**Emotional empathy**					
Diagnosis (AUTISM)	− 0.059 [− 0.255, 0.142]	0.121		0.057	0.594
Sex (F)	− 0.073 [− 0.275, 0.138]	0.135		0.072	0.544
Age group (Child/Adolescent)	0.025 [− 0.175, 0.214]	0.103		0.042	0.671
Valence (Negative)	− **0.647 [**− **0.80, **− **0.49]**	**1.61 × 10**^**–6**^		**4.89 × 10**^**5**^	**0.000**
Diagnosis × Valence	0.188 [0.066, 0.307]	3.521	5.59	1.075	0.076
**Arousal empathy**					
Diagnosis (AUTISM)	− 0.045 [− 0.232, 0.145]	0.104		0.046	0.653
Sex (F)	− 0.024 [− 0.234, 0.179]	0.110		0.048	0.640
Age group (Child/Adolescent)	0.084 [− 0.118, 0.289]	0.143		0.079	0.517
Valence (Negative)	− 0.137 [− 0.342, 0.071]	0.253		0.160	0.345
Age group x Valence	− **0.206 [**− **0.390, **− **0.149]**	**89.300**	**147**	**16.3**	**0.005**

Practically significant predictors for each empathy component are shown in bold.

**Figure 1:**
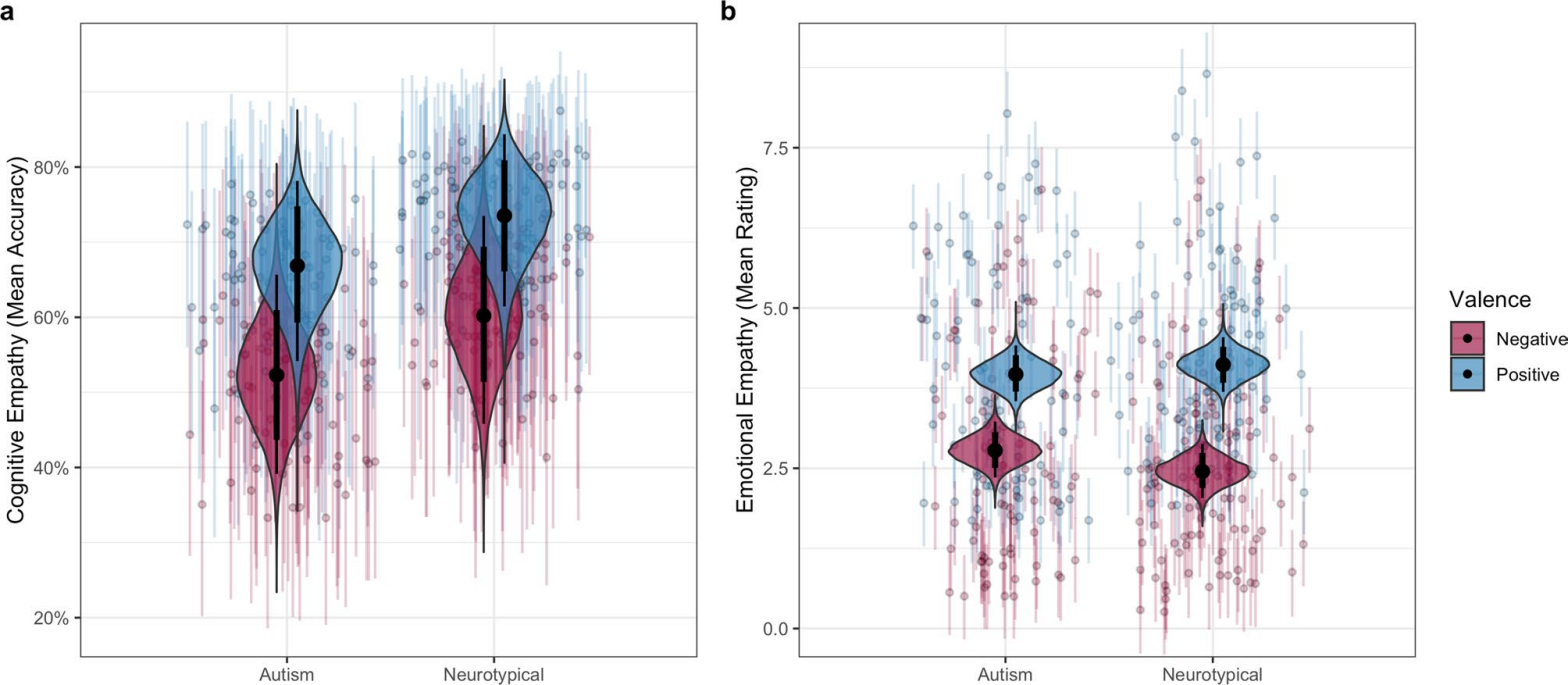
Group comparisons for (**a**) mean accuracy in emotion recognition for cognitive empathy surveys, and (**b**) mean resonance rating for emotional empathy surveys. Figure generated in R^41^.

In the best-fitting model, autism diagnosis was associated with a practically significant reduction in performance on cognitive empathy (CE) trials (OR = 0.726, 95% CrI [0.587, 0.906], *BF*_ROPE_ = 3.70). This effect of group on CE is depicted in Fig. 1A. Moreover, age group was an even larger predictor of performance, with children across both diagnostic groups displaying significantly lower emotion recognition accuracy than adults (OR = 0.604 [0.462, 0.795], *BF*_ROPE_ = 37.80). Neither female sex (OR = 1.113 [0.917, 1.352], *BF*_ROPE_ = 0.110) or negative valence (OR = 0.545 [0.267, 1.071], *BF*_ROPE_ = 1.609) significantly predicted performance on the cognitive empathy trials, although there was insufficient evidence to conclude that valence was unrelated to the chance of a correct response. Lastly, although higher VIQ significantly predicted higher performance on cognitive empathy trials (OR = 1.126 [1.037, 1.218] per standard deviation increase in VIQ), there was substantial evidence that this effect was too small to be practically significant (*BF*_ROPE_ = 0.213).

When predicting emotional empathy, model selection procedures indicated that the most likely model included all baseline predictors as well as the interaction between diagnosis and valence ($P\left({\mathcal{M}}_{BL+Dx*Valence}|Data\right)=0.452$; *R*^2^_Bayes_ = 0.505 [0.491, 0.518]). Inclusion Bayes factors supported only the inclusion of diagnosis × valence interaction term in the final model (*BF*_inc_ = 5.59), along with the exclusion of most other predictors (Table 1). The best-fitting model displayed a large effect of valence, with lower reports of shared feelings for negative emotions (β = − 0.647 [− 0.800, − 0.489], *BF*_ROPE_ = 4.89 × 10^5^), as well as small and practically insignificant main effects of diagnostic group (β = − 0.059 [− 0.255, 0.142], *BF*_ROPE_ = 0.057), sex (β = − 0.073 [− 0.275, 0.138], *BF*_ROPE_ = 0.072), and age group (β = 0.025 [− 0.175, 0.214], *BF*_ROPE_ = 0.042). The interaction between diagnosis and valence was statistically significant, although the ROPE Bayes factor was equivocal with regard to its practical significance (β = 0.188 [0.066, 0.307], *BF*_ROPE_ = 1.075). Although both diagnostic groups reported empathizing more with positive than negative emotions, this difference was larger in the NT group (*d* = 0.647 [0.489, 0.800]) than the autism group (*d* = 0.459 [0.315, 0.614]). This interaction is depicted in Fig. 1B.

**Figure 2:**
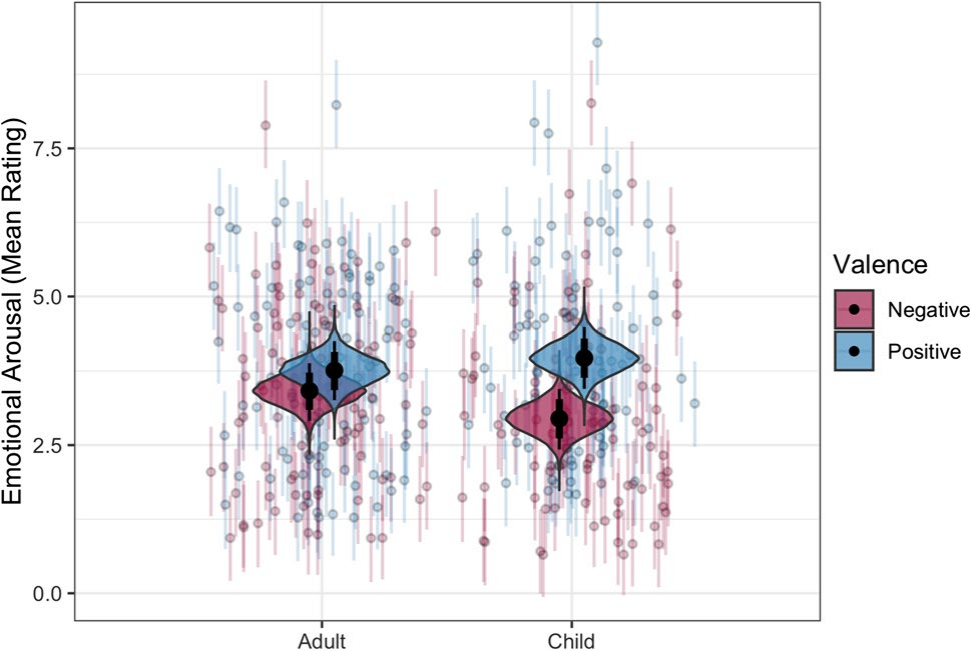
Age group comparisons for mean emotional arousal ratings to positive and negative emotionally charged facial expressions. Figure generated in R^41^.

When predicting ratings of arousal, the best-fitting model was found to include all baseline predictors as well as the interaction between age group and valence ($P\left({\mathcal{M}}_{BL+Age*Valence}|Data\right)=0.677$; *R*^2^_Bayes_ = 0.466 [0.451, 0.480]). Inclusion Bayes factors demonstrated strong support for the inclusion of the age group × valence interaction (*BF*_inc_ = 147), as well as the exclusion of all other potential predictors (Table 1). Coefficients in the best-fitting model indicated practically insignificant effects of autism diagnosis (β = − 0.045 [− 0.232, 0.145], *BF*_ROPE_ = 0.046), age (β = 0.084 [− 0.118, 0.289], *BF*_ROPE_ = 0.079), sex (β = − 0.024 [− 0.234, 0.179], *BF*_ROPE_ = 0.048), and emotional valence (β = − 0.137 [− 0.342, 0.071], *BF*_ROPE_ = 0.160). However, these effects were qualified by a statistically and practically significant interaction between age and valence (β = − 0.269 [− 0.390, − 0.149], *BF*_ROPE_ = 16.3). Across both diagnostic groups, children reported significantly higher arousal ratings for positive stimuli compared to negative stimuli (*d* = 0.403 [0.195, 0.609]), whereas no significant effect of valence was seen in adult participants (*d* = 0.137 [− 0.071, 0.342]). This interaction effect is illustrated in Fig. 2.

## Discussion

Using a multidimensional approach, this study replicates previous findings of practically equivalent levels of emotional empathy between autistic and NT groups despite a significant effect of diagnosis on cognitive empathy. These findings also complement Dziobek’s et al^26^, by extending the comparison to test for valence effects. To our knowledge, only one other group has assessed emotional valence effects in autism using the MET^27^, which reported that autistic adolescents displayed reduced emotional empathy for facial expressions of negative emotions compared to controls, a difference that was not present for positive emotions. Notably, the findings of Mazza and colleagues differ from those of the current study, which found that ratings of emotional empathy were approximately equal between groups for negatively-valenced stimuli, although neurotypical individuals provided higher average ratings for positively-valenced stimuli. The present study further extends Mazza’s et al^27^, results by assessing age group effects and utilizing a substantially larger child/adolescent autism sample. With regards to age, our Bayesian analysis indicates that children and adolescents across both diagnostic groups had greater difficulty with emotion recognition than adults, although main effects of age group on self-ratings of arousal and emotional empathy were small and practically insignificant.

Valence effects in our analyses were only significant in models predicting emotional empathy and arousal. For emotional empathy, the significant interaction between valence and diagnostic group indicated that positive facial expressions elicited greater emotional resonance than negative facial expressions across both groups, but that this difference was greater in neurotypicals compared to autistic individuals. In the arousal empathy Bayesian Model Averaging (BMA) analysis, the small and practically insignificant main effects for emotional valence and age group were qualified by a strong and practically significant interaction between these predictors. That is, the increase in arousal elicited by positive facial expressions relative to negative ones was more pronounced in our child and adolescent group compared to the adult group.

In the current study, we observed higher ratings for shared feelings to positive versus negative facial expressions that interacted with diagnostic group but not with age. This may reflect a neurotypical advantage for better self-other distinction for negative valence compared to autistic individuals that persists across age. In other words, contrary to previous reports of unequivocally intact emotional empathy in autism^26,42^, emotional empathy may actually differ somewhat in autism when valence is considered. The contrast between greater resonance to positive emotions compared to negative emotions across neurotypical development may also be partially attributable to the effect of positivity-biases for ambiguous emotions. In some neurotypical adults, ambiguous facial expressions like ‘surprise’ tend to get rated as positive, an effect that seems to be moderated by the regulatory influence of prefrontal cortex^43^. Thus, the lower degree of separation between emotional resonance ratings to positive and negative emotions in autism may also be reflecting neural differences in top-down emotion processing.

Supporting the cognitive vs. affective empathy dichotomy, Cox et al^18^ reported distinct intrinsic functional connectivity (FC) dynamics in healthy adult brains for self-reported cognitive and emotional empathy. Using a difference score (cognitive – emotional), this group found emotional empathy dominance (negative scores) to be associated with stronger functional connectivity between social-emotional brain regions like the amygdala and ventral anterior insula^18^. By contrast, cognitive empathy dominance (positive scores) correlated with greater FC in areas like the ventral anterior insula and superior temporal sulcus, both of which have been implicated in social-cognitive processes^18,44,45^. This distinction also becomes relevant when considering the implications of valence-specific evidence for empathy differences in autism like an impairment in the ability to understand when social experiences warrant resonating with negative emotions, a feature that has been linked to aggressive behaviors^46–48^.

The present study had various strengths and limitations to consider. Amongst the strengths, our study included a wide age range in which we were able to replicate previous findings from research conducted primarily in adolescent and adult samples, and extend findings to a larger group of younger individuals. Additionally, use of robust statistical approaches allowed us to report on the practical significance of our findings as well as to test for equivalence between diagnostic groups on these empathy measures. Ours is among the first to combine several important approaches in a single study: examining empathy multidimensionally, testing both children and adults, and examining positive and negative emotional valence separately. These advances facilitate a significant step forward in our understanding of empathy in autism.

This study was limited by exclusion of individuals with low IQ (FSIQ < 70) as a way of ensuring proper understanding of the task instructions. Future efforts to address this limitation should include objective psychophysiological empathy measures that do not require explicit behavioral response or abstract thinking. An additional limitation is the fact that the current study did not include measurements of trait alexithymia^49,50^, which has been proposed to mediate the relationship between diagnoses of autism and performance on tasks tapping multiple facets of empathy^17,51,52^. Another limitation is that we did not test for empathic concern and prosocial behavior propensity. In retrospect, we would have also liked to complement our findings by including at least one additional validated empathy questionnaire to compare and enrich our understanding on multimodal empathy assessment.

Within the context of social neuroscience, our measure of emotional empathy aligns more with indices of emotional accuracy which may be more relevant for generating social rapport compared to emotional concern and thus its relationship with prosocial behavior remains unexamined in this multidimensional way. Changes in the wording for indexing emotional empathy were modelled after the MET-J and pursued under the premise that the language used in the original MET may be too abstract for younger participants^28^. The implications of this change became apparent only after careful review of recent literature pointing to the differences between empathic accuracy and empathic concern^11^. Thus, future research designs ought to take this distinction into consideration and address as many of empathy components as possible within a single cohort in order to obtain a more holistic insight and understanding of empathy.

It should also be noted that a growing literature further improving ecological validity by utilizing dyadic conversational interactions (typically involving richer but less emotionally charged stimulation than the static but highly emotional faces used in the MET) has described a “double empathy” problem with assumptions made from empirical research in empathy and autism^53–55^. These reports critique the use of neurotypical people as a reference point and conclude that differences are diminished when autistic individuals are partnered with one another, suggesting that an empathy deficit (at least in the relatively emotionally neutral context of an initial conversation with a new person) should be reframed as a feature of the interaction rather than the individual^55^. Finally, The MET utilizes static emotional faces, which limits the ecological validity of the task. Following the example of recent studies that use dyadic interactions to characterize social differences in autism^55–57^, future studies should continue to balance ecological validity with experimental control under a framework that does not assume a neurotypical reference point.

The self-report nature in the design of the MET, albeit task-based, still confers some susceptibility to social desirability biases. This is one potential explanation for the increased emotional relatedness feelings reported by our neurotypical adult sample, who may be more impacted by social desirability bias^58^. The specificity of this difference to facial expressions depicting positive emotions and not negative emotions, however, warrants further investigation. Future efforts should aim to collect more implicit measures of emotional empathy such as skin conductance, spontaneous facial expressions, and neural measures of empathic response. Within the context of conflated definitions and methodologies employed in previous research, the resulting confusion from conflicting academic reports on the autistic ‘empathy deficit’ has not been without effect on the autistic population. Overgeneralizations on this matter have been described as ‘unwarranted’ and ‘dehumanizing’ by autistic self-advocates^59^. Because of this negative impact potential, future work should also take great care to develop methodologies based on clearly defined empathy concepts as well as reporting and interpreting results using a more humane framework.

In conclusion, the current study finds component-specific differences in empathy. We report impaired cognitive empathy in autism, a valence by diagnostic group effect in emotional empathy, and valence by age group effect for arousal empathy. In both diagnostic groups, emotion recognition as measured by our cognitive empathy survey was significantly greater in adults than in children and adolescents. Improved emotion recognition by adulthood across groups may potentially reflect training effects of social interventions in autism and a larger repertoire of references for positive interactions in neurotypicals. Further investigations would benefit from an analysis that accounts for potential confounds like co-occuring mental health symptoms or training effects on the ability of individuals with autism to recognize emotions. These findings suggest that emotional stimuli, specifically hedonically negative stimuli, may be actively recruiting separate perceptual pathways that are differentially altered in autism. Better elucidating component-specific impairments is crucial for the generation of target-specific social interventions to improve empathic capacities and social outcomes.

## Methods

### Participants

Participants in this study were recruited from the community through posters and social media postings as well as from a pool of participants in previous larger and longitudinal lab studies who consented to be recontacted. Over the course of the recruitment period, our stance on study design for case–control comparisons has evolved; early in this period, the goal was to achieve a “clean” ASD sample and thus excluded for most co-occurring psychiatric and developmental conditions within the ASD group. As time has gone by, we have seen that these samples are not representative of the population, and have opted to be more inclusive and attempt to control for co-occurring conditions in both groups analytically instead, though we have been slower to adopt this for control groups. Thus, this sample represents a blend as our approach has evolved.

Co-occurring mental health symptoms in our autism group were screened for using the ASEBA School Age (6–18) & ASEBA Adult (18–59) forms. Overall, we collected ASEBA data on 90/184 participants (24 ASD, 66 NT). Of these, 11 ASD participants endorsed clinically significant symptoms of depression, 8 endorsed clinically significant symptoms of anxiety, and 9 endorsed clinically significant symptoms of ADHD. Of the 66 NT participants with ASEBA data, 3 endorsed clinically significant symptoms of depression, and 1 participant endorsed clinically significant symptoms of ADHD.

Individuals with autism and co-occurring ADHD, anxiety, or depression were included, while those with other psychiatric diagnoses within the past five years or co-occurring neurogenetic syndromes were excluded. Stimulant medication use was screened for before study participation but there was no exclusionary criteria based on this. Of our 184 participants, 36 endorsed ‘Yes’ to taking medications and of these, 11 (all ASD) reported using stimulant medication. In our adult subgroup, ten autistic adults (26%) reported taking antidepressant medications, and in our children/adolescents subgroup, ten autistic participants’ parents (22%) reported their child taking antidepressant medications.

### Adults

Thirty-eight adult participants with autism (21 male; mean age = 27.65) and 58 neurotypical (NT) adults (36 male; mean age = 32.69) were included in the study. All adult participants were between the ages of 18 and 59 years and achieved full scale IQ scores of $\ge$ 70 as measured by the Wechsler Abbreviated Scale of Intelligence-Second Edition^60^ (WASI-II). Autism diagnoses were confirmed by the clinical judgment of a licensed psychologist specializing in the assessment of autism, supported by research-reliable administration of the Autism Diagnostic Observation Schedule-2^61^ (ADOS-2).

Exclusion criteria for both groups included the presence of other neurological and genetic disorders, non-autism related sensory impairments (e.g., uncorrected visual or hearing impairments), and substance/alcohol abuse or dependence during the past two years. Further, individuals in the NT group were excluded if they had reported a previous psychiatric history, cognitive or sensory impairment, use of psychotropic medications, or clinically elevated scores on the Social Communication Questionnaire^62^ (SCQ Total score > 15).

### Children/adolescents

Forty-five autistic children/adolescents (35 male; mean age = 11.53) and 43 neurotypical (NT) children/adolescents (34 male; mean age = 11.86) were included in the study. All child/adolescent participants were between the ages of 8 and 17 years and achieved full scale IQ (FSIQ) scores of $\ge$ 70 as measured by the WASI-II. Autism diagnoses were confirmed by the clinical judgment of a licensed psychologist specializing in the assessment of autism, supported by research-reliable administration of the ADOS-2 and, when available, parent interviews (n = 30) that included algorithm items from the Autism Diagnostic Interview, Revised^63^ (ADI-R).

Exclusion criteria for children/adolescents were similar to those for adults with some additional considerations. Mainly, for children and adolescents, behavior and co-occurring psychiatric conditions were screened for using parent and guardian reports.

### Ethical considerations

The study was conducted in accordance with the Declaration of Helsinki and all participants were compensated $20 per hour of their time following each session. Written informed consent or assent forms were signed by all participants, while informed consent was obtained from parents or guardians of minors. All methods and procedures were approved by the Institutional Review Board for human subjects at Vanderbilt University Medical Center and carried out in accordance with relevant guidelines and regulations on ethical human research.

### Measures

The Social Responsiveness Scale–Second Edition^64^ (SRS-2) was used to measure autistic traits dimensionally across the full sample. Adult participants in both diagnostic groups completed the SRS-2 adult self-report form, whereas parents or guardians of children/adolescents in both groups completed the analogous caregiver-report questionnaire, the SRS-2 School Age form. To facilitate comparison across the different groups, the SRS-2 total scores were converted to T- scores (*M* = 50, *SD* = 10).

### Empathy

**Figure 3:**
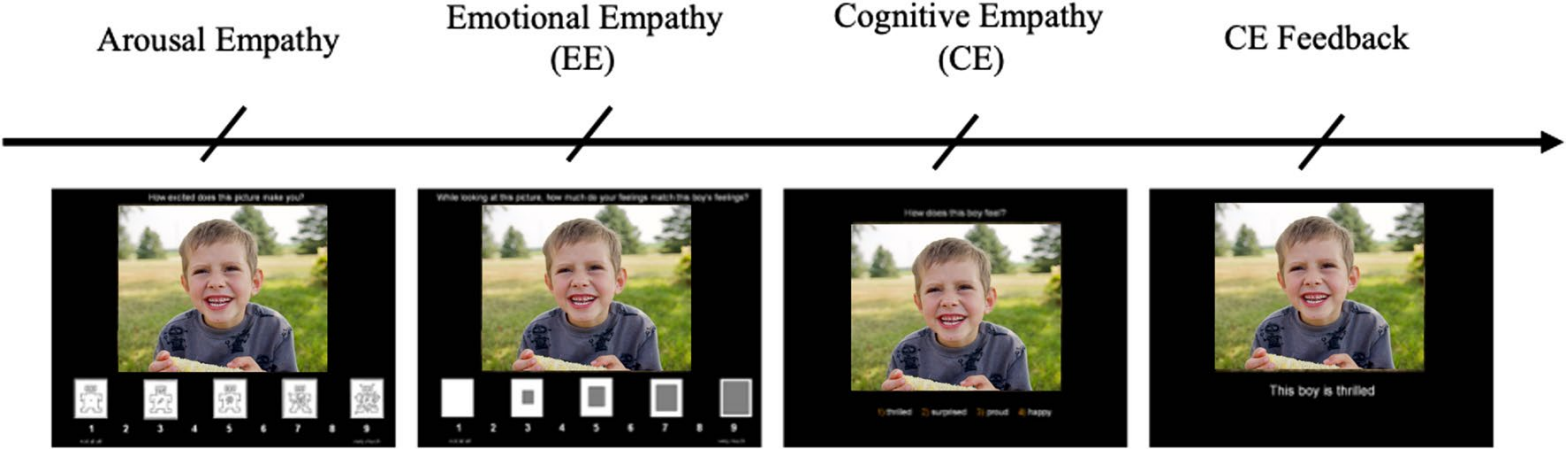
Example trial for ‘thrilled’ emotion depicted on MET-J task. The surveys read 1) “How excited does this picture make you” (implicit emotional empathy/arousal empathy), 2) “While looking at the picture, how much do your feelings match the boy’s feelings (emotional empathy; EE, and 3) “How does this boy feel?” (cognitive empathy; CE). Cognitive empathy emotion label options are 1) thrilled, 2) surprised, 3) proud, 4) happy. The slides for this trial example were designed on PowerPoint using a free-use stock image from the Canva.com image database.

Empathy was assessed multi-dimensionally using an adapted version of the Multifaceted Empathy Test, the MET-J^28^, a validated performance-based test that separates cognitive and emotional empathy based on responses to emotional faces presented with context in the background. The original MET includes 50 still images depicting emotionally charged facial expressions of 25 positive (e.g., joy, happiness) and 25 negative (e.g., sadness, anger) emotions. The adapted MET-J version used in the present study included only 16 images each for positive and negative valence. The photographs are taken from the International Affective Picture System^65^ (IAPS), a well-validated database of photographs designed for standardized emotion and attention testing. On each trial, participants viewed an emotional image and were first asked to rate their level of arousal, followed by explicit emotional empathy ratings, and a cognitive empathy (i.e., emotion recognition) multiple choice question. Figure 3 depicts an example trial on the task, recreated using a free-use stock image from the Canva.com image database.

As described by Dziobek et al. (2008), to minimize demands of self-reflection and thereby also mitigate social desirability bias, we included an implicit assessment of emotional empathy by asking participants to rate how calm/aroused the emotional stimuli made them feel using the Self-Assessment Manikin (SAM). The SAM is a visual-analogue scale providing scores ranging from 1 (very calm) to 9 (very aroused). Thus for each picture, participants were asked (1) “How excited does this picture make you” (implicit emotional empathy; subsequently described as arousal empathy); (2) “While looking at the picture, how much do your feelings match the X’s feelings” (emotional empathy; EE) measured on a visual Likert scale (1–9); and finally (3) “How does this X feel?” (cognitive empathy; CE). Here an “X” represents the noun used to describe the individuals (boy/girl/man/woman) in the image, who varied across trials. Each trial ended with a final presentation of the emotional stimulus that provided feedback for the cognitive empathy question by displaying the correct emotion label from among the four choices. Note that this order and wording for EE surveys are slightly different from the original MET and MET-J, which provided feedback on CE surveys prior to presenting explicit emotional empathy surveys. We adapted this order to ensure that EE and arousal responses were made as reflexively as possible to the perceived emotion upon initial presentation, rather than being adjusted based on CE feedback. All stimuli were presented as slides of variable duration (ad libitum) in random order on a black screen.

### Statistical analyses

Differences in demographics (e.g., age, sex, VIQ, PIQ) and SRS-2 scores were compared between the autism and NT groups within a Bayesian framework. When the outcome of interest was categorical (e.g., correct or incorrect emotion recognition), group differences were examined using a Bayesian analogue of the Pearson chi-squared test^66,67^. When the outcome of interest was a continuous variable (e.g., age), we examined mean differences using a Bayesian analogue of the Welch (unequal-variances) *t*-test^68^. Effect sizes from each of these tests (i.e., Cohen’s *d* and the odds ratio [OR]) were summarized as the posterior median and 95% highest-density credible interval (CrI). Additionally, for all group comparisons, evidence for or against the point null hypothesis (${\mathcal{H}}_{0}$; i.e., no differences between groups) was quantified with a Bayes factor^67,69^, defined as the ratio of how likely the data are under the alternative hypothesis (${\mathcal{H}}_{1}$; i.e., the difference between group is nonzero) divided by how likely the data are under ${\mathcal{H}}_{0}$. In concordance with widely-used guidelines on Bayes factor interpretation^70,71^, we considered *BF*_10_ values > 3 as indicating substantial evidence for ${\mathcal{H}}_{1}$, *BF*_10_ values < 0.333 as indicating substantial evidence for ${\mathcal{H}}_{0}$, and *BF*_10_ values between 0.333 and 3 as providing inconclusive and only “anecdotal” evidence for ${\mathcal{H}}_{0}$ or ${\mathcal{H}}_{1}$. All group comparisons were performed in the R statistical computing platform using open-source R code written by author ZJW^72^. Additional details on the specifics of the models underlying Bayesian *t*-test and Chi-squared test analogues are presented in “Supplemental Methods”.

To determine the effects of various predictor variables on arousal, emotional, and cognitive empathy while controlling for possible covariates, we used R^41^ to analyze the data at the single-trial level using hierarchical Bayesian modeling. Trial-level MET data for arousal, emotional, and cognitive empathy were analyzed using (generalized) linear mixed effects models ([G]LMEMs), which allowed us to model the correlations between responses derived from the same participants as well as the same stimuli^73^. LMEMs were used to model arousal and emotional empathy, as the 9-item scale used to derive these outcomes had enough points to be approximated as a continuous variable^74^. However, we used a logistic GLMEM to model cognitive empathy, as individual trial data from this part of the task consisted of binary “correct/incorrect” responses. The baseline [G]LMEM for each MET-derived outcome included fixed effects of age group (child vs. adult), sex, autism diagnosis, and emotional valence (positive vs. negative), as well as random intercepts for participant and stimulus (see example below for CE, Eq. 1). Random slopes were also included in this baseline model for all subject-level predictors, allowing the effects of age group, sex, and autism status to vary by stimulus. The decision to treat age as categorical in the BMA was driven by the finding that performance on the CE task increased with age throughout childhood, reaching an asymptote at approximately age 18–20, thereby indicating a difference between children and adults rather than a true linear age trend.

1 $$\text{Cognitive Empathy Dx Group} + \text{Age Group}  + \text{Sex}  +  \text{Valence}  +  (1\text{|Participant)}  + (1  +  \text{Stimulus |Dx Group}  +  \text{Sex}  +  \text{Age Group)}$$

For each of the three outcomes, we additionally determined if several other predictors beyond the baseline model contributed to task performance, including the two-way and three-way interactions between age, diagnosis, and valence; verbal IQ (VIQ); performance IQ (PIQ); and overall level of autistic traits (SRS-2 T-score). In order to determine whether any given predictor should be added to the baseline model, we fit candidate models that included all combinations of potential predictors (*n* = 40 potential models including the baseline). Then, using bridge sampling^75^, we calculated marginal likelihood of each candidate model, deriving posterior model probabilities in a manner equivalent to the process of Bayesian model averaging^76^. The model with the highest posterior probability was considered the final model for each outcome. Using these model weights, we also computed inclusion Bayes factors^76^ (*BF*_inc_), allowing us to determine the degree of evidence for or against the inclusion of each predictor in the model. Inclusion Bayes factors are interpretable in the same manner as *BF*_10_, with ${\mathcal{H}}_{0}$ being the exclusion of the variable from the model and ${\mathcal{H}}_{1}$ being the inclusion of the variable in the model.

Once the final model for each outcome was selected, we additionally tested all regression slopes in a Bayesian framework, using the 95% CrI to determine whether each slope was likely to be nonzero in magnitude. If the full 95% CrI excluded zero, we rejected the point null hypothesis that the effect was exactly zero. However, as this point null hypothesis is always false at the population level^77^, we also tested these effects for *practical* significance^78^. The Bayesian framework allows for a probabilistic view of the parameter estimates so that we can infer whether an effect is practically meaningful at the population level. This is done by defining a region of practical equivalence^79^ (ROPE), an interval of parameter values considered small enough to be equivalent to zero in practice (in this case ${\upbeta }_{\mathrm{Std}}=\left[-\mathrm{0.1,0.1}\right]$ for linear models and ${e}^{{\upbeta }_{Std}}=\left[\mathrm{0.909,1.10}\right]$ for logistic models). Evidence both for and against the true parameter value falling within the ROPE can be quantified by calculating a ROPE Bayes factor (*BF*_ROPE_), defined as the odds of the prior parameter distribution falling within the ROPE divided by the odds of the posterior effect size distribution falling within the ROPE^80,81^. These Bayes factors can be interpreted on the same scale as previously discussed for *BF*_10_ *and BF*_inc_^70,71^. In the case that a parameter was nonzero or a given variable was included within the final model but the *BF*_ROPE_ value was smaller than 0.333, we considered this variable as not predicting the MET outcome of interest to a practically meaningful extent. Lastly, in order to assess the predictive power of the final model, we calculated the Bayesian *R*^2^ coefficient proposed by Gelman et al.^82^.

All Bayesian [G]LMEMs were fit in Stan using the *brms* R package^83,84^ with weakly informative priors, including Normal(0, 1) priors on all (standardized) regression slopes and intercept terms, as well as default half-Student *t*_3_(0, 2.5) priors on the standard deviation of each random slope or intercept term. Model parameters were estimated via Markov chain Monte Carlo (MCMC) using the No U-turn Sampler implemented in Stan^85^, with posterior distributions of each parameter estimated using 21,000 post-warmup MCMC draws from seven Markov chains (14,000 in cases where missing data were present). Parameter summaries from these posterior distributions were operationalized as the posterior median and the 95% CrI. Convergence for each model was confirmed by examination of Markov chain trace plots, as well as values of the Gelman–Rubin (Rubin & Gelman, 1992) convergence diagnostic < 1.01. Missing data were handled using five-fold multiple imputation based on the random forest imputation algorithm implemented in the *missForest* R package^86,87^.

## Supplementary Information

Supplementary Information.

## Data Availability

The datasets generated for this study can be found in the National Database for Autism Research (NDAR) repository (https://nda.nih.gov).
