# Supplementary material for: Multifaceted empathy differences in children and adults with autism
Source: Sci Rep. 2021 Sep 30;11:19503. doi: 10.1038/s41598-021-98516-5 (PMC8484273; doi:10.1038/s41598-021-98516-5)
Supplement: Supplementary file 1 — Supplementary Information. [file 41598_2021_98516_MOESM1_ESM.docx]

**Supplemental Methods**

### **Bayesian Unequal-variance *t*-tests**

All statistical computations were performed in R^1^. When comparing continuous variables between autistic and neurotypical groups, we utilized a Bayesian *t*-test similar to the BEST procedure proposed by Kruschke^2^. However, a notable difference is that in our model, priors were not dependent on the data, dependent variables were standardized (*M* = 0, *SD* = 1). The standardized outcome was fit to an unequal-variances *t*-test model in the *brms* R package^3^, with a Normal(0, 1) prior on regression coefficients (i.e., the intercept term, mean difference between groups, and mean difference in log(σ) between groups), a Normal(0, 1) prior on log-transformed standard deviation parameters for each group, and a Gamma(2, 0.1) prior on ν, the degrees of freedom of the *t-*distribution. Posterior distributions of the parameters were based on 40,000 post-warmup MCMC draws from five Markov chains. The primary parameter of interest was the standardized mean difference between groups (i.e., Cohen’s *d*, calculated as the difference in means divided by the square root of the pooled variance), which we summarized using the posterior median and 95% highest-density credible interval (CrI). The Savage-Dickey density ratio^4^ was used to calculate a Bayes factor testing the hypothesis that *d* = 0.

### **Bayesian Chi-squared Tests**

Gender was compared between the autistic and neurotypical groups using the Bayesian equivalent of a Chi-squared test, which assessed the hypothesis that the proportion of females in the two groups was equal. The proportions of females in each group were compared using default Gûnel-Dickey Bayes factors for contingency tables (as implemented in the BayesFactor R package^5^) based on the independent multinomial sampling scheme^6,7^. In this test, the number of individuals in each diagnostic group (autistic and neurotypical) is treated as fixed, and cell counts are multinomially distributed within each row of the contingency table. A Dirichlet prior with parameters $\alpha_{1},\ldots,\alpha_{k}=1$ is placed on the parameters of each multinomial distribution, and the analytically derived Bayes factor provides evidence for or against the null hypothesis of equivalent distributions between groups. In addition, we calculated the odds ratio (OR) along with its 95% CrI using 15,000 Monte Carlo samples from the joint posterior distribution of the model parameters.

**Supplemental References**

1. R Core Team. *R: A Language and Environment for Statistical Computing*. (R Foundation for Statistical Computing, 2021).

2. Kruschke, J. K. Bayesian estimation supersedes the t test. *J. Exp. Psychol. Gen.* **142**, 573–603 (2013).

3. Bürkner, P.-C. brms: An R package for Bayesian multilevel models using Stan. *J. Stat. Softw.* **80**, 1–28 (2017).

4. Wagenmakers, E.-J., Lodewyckx, T., Kuriyal, H. & Grasman, R. Bayesian hypothesis testing for psychologists: A tutorial on the Savage–Dickey method. *Cognit. Psychol.* **60**, 158–189 (2010).

5. Morey, R. D. & Rouder, J. N. *BayesFactor: Computation of Bayes Factors for Common Designs*. (2018).

6. Gûnel, E. & Dickey, J. Bayes factors for independence in contingency tables. *Biometrika* **61**, 545–557 (1974).

7. Jamil, T. *et al.* Default “Gunel and Dickey” Bayes factors for contingency tables. *Behav. Res. Methods* **49**, 638–652 (2017).
